# Impact of disulfidptosis-associated clusters on breast cancer survival rates and guiding personalized treatment

**DOI:** 10.3389/fendo.2023.1256132

**Published:** 2023-12-05

**Authors:** Xiong Chen, Guohuang Hu, Qianle Yu

**Affiliations:** Department of General Surgery, Affiliated Changsha Hospital of Hunan Normal University, Changsha, China

**Keywords:** breast cancer, disulfidptosis, single-cell, immunotherapy, prognosis

## Abstract

**Background:**

Breast cancer (BC) poses a serious threat to human health. Disulfidptosis is a recently discovered form of cell death associated with cancer prognosis and progression. However, the relationship between BC and disulfidptosis remains unclear.

**Methods:**

We integrated single-cell sequencing and transcriptome sequencing in BC to assess the abundance and mutation status of disulfidptosis-associated genes (DAGs). Subsequently, we clustered the samples based on DAGs and constructed a prognostic model associated with disulfidptosis. Additionally, we performed pathway enrichment, immune response, and drug sensitivity analyses on the model. Finally, we validated the prognostic genes through Immunohistochemistry (IHC).

**Results:**

The single-cell analysis identified 21 cell clusters and 8 cell types. By evaluating the abundance of DAGs in different cell types, we found specific expression of the disulfidoptosis core gene SLC7A11 in mesenchymal stem cells (MSCs). Through unsupervised clustering of DAGs, we identified two clusters. Utilizing differentially expressed genes from these clusters, we selected 7 genes (AFF4, SLC7A11, IGKC, IL6ST, LIMD2, MAT2B, and SCAND1) through Cox and Lasso regression to construct a prognostic model. External validation demonstrated good prognostic prediction of our model. BC patients were stratified into two groups based on riskscore, with the high-risk group corresponding to a worse prognosis. Immune response analysis revealed higher TMB and lower TIDE scores in the high-risk group, while the low-risk group exhibited higher CTLA4/PD-1 expression. This suggests that both groups may respond to immunotherapy, necessitating further research to elucidate potential mechanisms. Drug sensitivity analysis indicated that dasatinib, docetaxel, lapatinib, methotrexate, paclitaxel, and sunitinib may have better efficacy in the low-risk group. Finally, Immunohistochemistry (IHC) validated the expression of prognostic genes, demonstrating higher levels in tumor tissue compared to normal tissue.

**Conclusion:**

Our study has developed an effective disulfidptosis-related prognostic prediction tool for BC and provides personalized guidance for the clinical management and immunotherapy selection of BC patients.

## 1 Introduction

BC remains one of the most common cancers in women with a high mortality rate (1). In the past, treatments such as mastectomy, radiation therapy, and chemotherapy have shown good efficacy in systemic management (2). With the advancements in modern medicine, traditional surgery is no longer the optimal choice for all patients. Targeted therapy, endocrine therapy, and immunotherapy are emerging as mainstream adjuvant treatments (3). However, the prognosis for BC patients remains poor, especially for triple-negative breast cancer (TNBC) and advanced-stage metastatic breast cancer (4, 5). In this situation, further exploration of the biological mechanisms of BC, improvement of early detection rates, and enhancing prognosis become crucial.

Programmed cell death (PCD) refers to the self-destruction of human cells to maintain internal stability (6). It is regulated by biomolecules and differs from accidental cell death (ACD) (7). PCD can occur through three main forms: apoptosis, pyroptosis, and necroptosis (8). The importance of cell death in cancer therapy has been recognized in recent years. Various forms of cell death, such as cuproptosis, pyroptosis, necroptosis, and ferroptosis, have been extensively studied (7). Recently, Liu et al. discovered that high expression of SLC7A11 induces a novel form of cell death distinct from apoptosis and ferroptosis (9). The significant accumulation of cystine is highly toxic to cells, compelling cancer cells with high levels of SLC7A11 to reduce cystine to cysteine, resulting in substantial consumption of nicotinamide adenine dinucleotide phosphate (NADPH) (10). During glucose deprivation, the pentose phosphate pathway, responsible for intracellular NADPH production, is impeded, leading to intracellular disulfide accumulation and rapid cell death (11). Liu et al. termed this new form of cell death disulfidptosis and proposed its significant potential in cancer therapy. It is noteworthy that Carlisle et al. suggested a close association between BC and SLC7A11 through selenium (12). Xu et al. found, through immunofluorescence, that the expression of SLC7A11 in BC tissues was significantly higher than in adjacent tissues (13). Given the strong correlation between SLC7A11 and disulfidptosis, disulfidptosis likely represents a new opportunity in BC treatment. However, research on the relationship between BC and disulfidoptosis is currently limited, and the potential biological mechanisms remain unclear. Clarifying their relationship would be of great assistance in prognosis prediction and treatment selection for BC patients.

The purpose of this study is to investigate the impact of disulfidptosis on the treatment and prognosis of BC. We assessed the abundance of DAGs in BC single-cell sequencing and transcriptome sequencing, constructed a prognosis model associated with disulfidptosis, and investigated the sensitivity of this model to immunotherapy and chemotherapy drugs. Through validation, our model demonstrated good accuracy, which could potentially offer insights for personalized treatment in BC.

## 2 Original research

## 3 Methods

### 3.1 Obtain BC sequencing data and DAGs

Transcript expression data for BC patients were obtained from The Cancer Genome Atlas (TCGA) and Gene Expression Omnibus (GEO) databases. The TCGA-BRCA dataset with gene expression and clinical information was selected from the TCGA database. Screening of three BC patient datasets (GSE20685, GSE58812, and GSE88770) using expression profiles and survival information from the GEO database was conducted. Because they were both based on the GPL570 platform, the Sva package was used to remove batch effects. The TCGA-BRCA dataset will be used as the training cohort and the combined-GSE dataset will be used as the external validation cohort. The single-cell data was obtained from GSE176068, where Single-cell RNA sequencing (scRNA-seq) was performed on 26 primary tumor tissues representing three BC subtypes (ER+, HER2+, and TNBC) (14). This dataset provided a comprehensive transcriptional atlas of BC cell structures, and its reliability has been substantiated by numerous studies (15, 16). Additionally, 24 disulfidptosis-associated genes, including FLNA, FLNB, MYH9, TLN1, ACTB, MYL6, MYH10, CAPZB, DSTN, IQGAP1, ACTN4, PDLIM1, CD2AP, INF2, SLC7A11, SLC3A2, RPN1, NCKAP1, NCKAP1, NUBPL, NDUFA11, LRPPRC, OXSM, NDUFS1, and GYS1, were obtained from Liu’s research (9).

### 3.2 Processing and analysis of scRNA-seq

The Seurat software package was utilized for reading and transforming scRNA-seq data (17). The quality control (QC) criteria were as follows: 1) use the PercentageFeatureSet package to calculate the percentages of mitochondria, ribosomes, and hemoglobin to exclude low-quality cells; 2) exclude genes detected in <3 cells; 3) using the FindVariableFeatures function to screen the top 2000 highly variable genes. Dimensionality reduction was performed using Uniform Manifold Approximation and Projection (UMAP) after Principal Component Analysis (PCA) (18). Additionally, distinct cell clusters were annotated using the singleR package (19). The VlnPlot and featureplot functions were adopted to characterize the abundance of DAGs in different cells.

### 3.3 Mutation profiling, differential correlation, protein-protein interaction analysis

Mutation data were downloaded from the TCGA database to perform a somatic mutation waterfall plot of the DAGs. The PPI network of DAGs was constructed using web tools (http://genemania.org/). To explore whether there are differences in the expression of DAGs between tumor and normal tissues, we conducted differential analysis and assessed the correlation among DAGs.

### 3.4 Consistent unsupervised clustering of DAGs

We conducted an expression matrix-based consensus clustering analysis of 24 genes associated with disulfidptosis using the ConsensusClusterPlus function (20). Survival analysis based on clusters and a heatmap analysis were carried out by incorporating clinical features. Furthermore, we performed an immune infiltration analysis using the CIBERSORT package (21).

### 3.5 Variance analysis, prognostic modeling, validation of external data

The limma package was used to perform differential analysis on the training cohort (22). Cox and Lasso regression were used to reduce the dimensionality of differentially expressed genes, and seven genes were identified. The surface equation of the prediction model was constructed as follows: riskscore = b1m1+ b2m2+ b3m3 … bNmN. Here, “b” represents the coefficient, “m” denotes gene expression, and “n” is the ordinal number of the prognosis-related genes. The average riskscore was used to classify samples into high-risk and low-risk groups. Kaplan-Meier survival curves and ROC curves were utilized to evaluate the predictive ability of the prognostic model for overall survival (OS). Univariate and multivariate Cox regression analyses combined with clinical characteristics were performed to identify risk factors. Additionally, the model was validated using the external cohort.

### 3.6 Nomogram construction and test, correlation of riskscore with clinical factors and prognostic analysis

We aimed to construct a nomogram to enhance the clinical value of the disulfidptosis-related prognostic model. Calibration curves and decision curve analysis (DCA) were used to assess the predictive performance and clinical application of our model. In addition, we evaluated whether there were differences in riskscore corresponding to different clinical features and conducted a survival analysis.

### 3.7 Analysis of biological mechanism pathways enrichment

To further explore the biological mechanisms of disulfidptosis, Genome Oncology (GO) and Kyoto Encyclopedia of Genes and Genomes (KEGG) analyses were performed using the ClusterProfiler software package (23), GSEA analysis was conducted based on h.all.v7.1. symbols.gmt.

### 3.8 Immune escape and chemotherapy sensitivity prediction

The Tumor Immune Dysfunction and Exclusion (TIDE) website was used to calculate the immune evasion status for these two risk groups. Predicting chemotherapy drug sensitivity using the pRRophetic software package (24).

### 3.9 Immunohistochemical validation of prognosis-related genes

To validate our screening for prognosis-related genes, we performed a search on the Human Protein Atlas (HPA) website, yielding IHC results corresponding to the tumor tissue and adjacent non-tumor tissue (https://www.proteinatlas.org/).

### 3.10 Statistics

All statistical calculations were performed by R 4.2.2 and SPSS 13.0. The t-test was used for normally distributed variables, and the Wilcoxon test was employed for non-normally distributed variables. Correlation was evaluated by Spearman analysis, and p<0.05 was considered statistically significant.

## 4 Results

### 4.1 Single-cell RNA clusters annotation and DAGs expression between different cells

**Figure 1:**
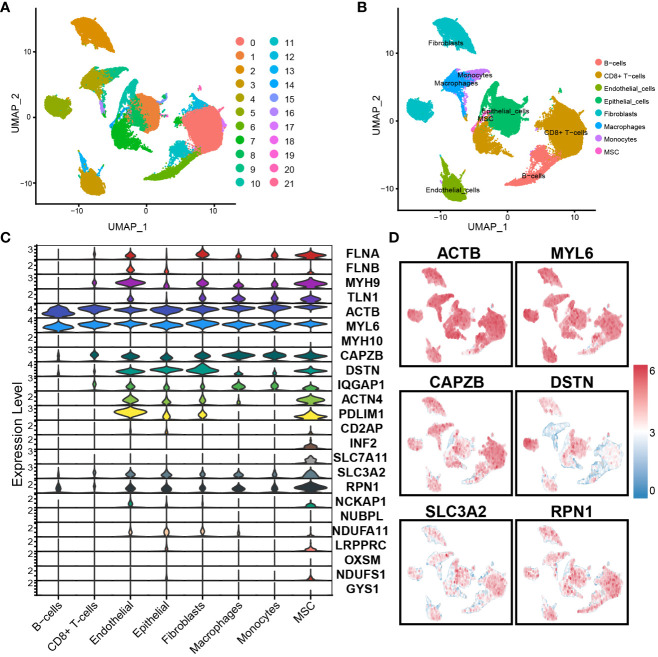
Single-cell RNA sequence analysis: **(A)** The dataset is divided into 21 clusters. **(B)** Cells were annotated into 8 significant cell populations. **(C)** Expression of DAGs in different cell populations. **(D)** Expression of important genes.

We used the scRNA-seq dataset GSE176078 from BC patients to examine the expression of 24 DAGs. Through analysis using R software, the dataset was divided into 21 cell clusters and 8 significant cell populations (**Figures 1A, B**), namely B cells, Endothelial cells, CD8+ T cells, Monocytes, Fibroblasts, Epithelial cells, Macrophages, and MSCs. Furthermore, gene expression was performed on the DAGs, revealing varying expression levels of each gene across different cell types. Among them, ACTB and MYL6 exhibited the highest expression levels. ACTB, MYL6, CAPZB, DSTN, SLC3A2, and RPN1 were expressed across all 8 cell populations (**Figures 1C, D**). Interestingly, SLC7A11 exhibits specific expression in MSCs, indicating the potential presence of some unknown connection between MSCs and disulfidptosis in BC.

### 4.2 Somatic mutations, PPI network, expression of DAGs

**Figure 2:**
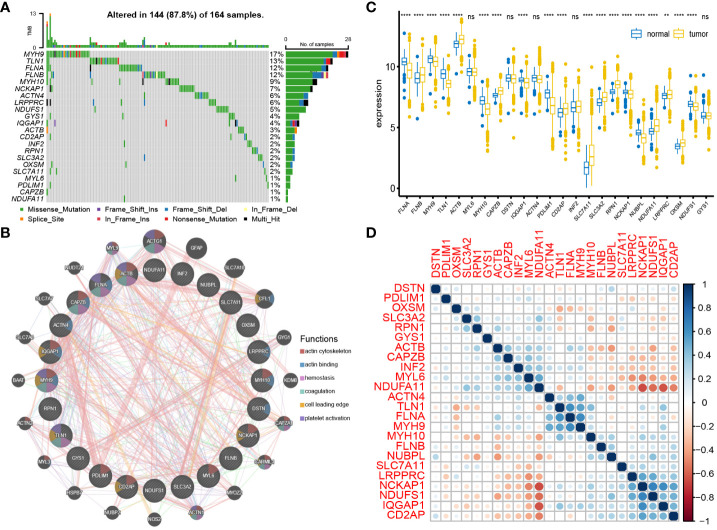
Interaction and mutation of DAGs: **(A)** Mutation waterfall plot of 24 DAGs. **(B)** PPI network of DAGs. **(C)** Differential expression of DAGs between tumor tissues and normal tissues. **(D)** Correlation analysis between DAGs. **P < 0.01; ****P < 0.0001; ns, no significance.

The waterfall plot of the DAGs in the TCGA cohort revealed that out of 164 samples, 144 (87.8%) had mutations. The mutation rates for MYH9, TLN1, FLNA, and FLNB were all above 10%. The most common type of mutation observed was a missense mutation (**Figure 2A**). The PPI network of DAGs was derived from the GeneMANIA website, and ACTB, FLNA, CAPZB, MYH9, and TLN1 were identified as hub genes in this network (**Figure 2B**). The differential analysis DAGs between tumor and normal tissues revealed overexpression of FLNB, ACTB, CAPZB, CD2AP, SLC7A11, SLC3A2, RPN1, NDUFA11, LRPPRC, and OXSM in tumor tissues; FLNA, MYH9, TLN1, MYH10, IQGAP1, PDLIM1, NCKAP1, NUBPL, and NDUFS1 were overexpressed in normal tissues (**Figure 2C**). This further confirmed the overexpression of the core disulfidptosis gene SLC7A11 in BC. Additionally, hub genes exhibited differential expression between cancer and non-cancer tissues, suggesting the significant potential of disulfidptosis as a novel therapeutic approach for BC. Correlation analysis of DAGs revealed that FLNA had the highest positive correlation coefficients with TLN1 and MYH9, while NDUFA11 had the highest negative correlation coefficients with NCKAP1 and IQGAP1. Importantly, the correlation of SLC7A11 with other DAGs was not strong. (**Figure 2D**)

### 4.3 Consensus clustering and immune cell infiltration

**Figure 3:**
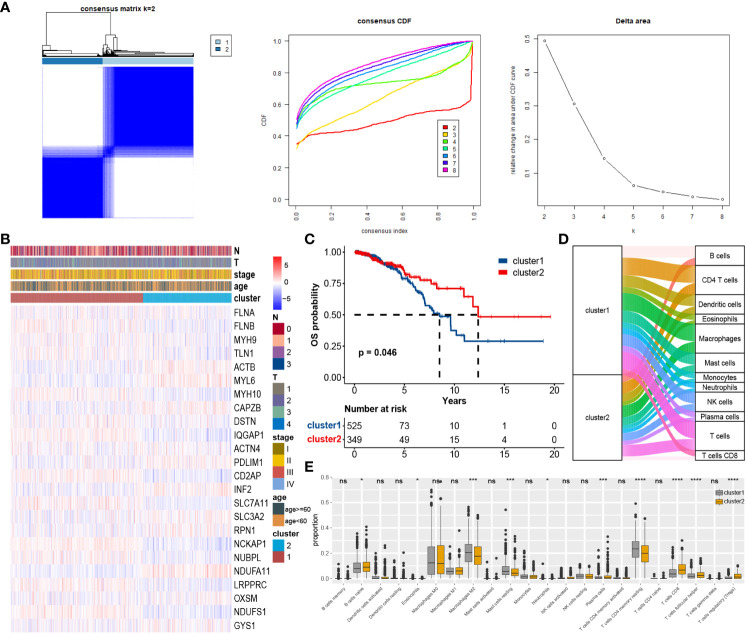
Consensus unsupervised clustering based on DAGs: **(A)** BC patients were divided into two clusters. **(B)** Heatmap of clusters and clinical features. **(C)** Survival analysis of two custers, cluster 1 corresponds to worse prognosis. **(D)** Immune cell infiltration between clusters. **(E)** Differential expression of individual immune cells among clusters. *P < 0.05; ***P < 0.001; ****P < 0.0001; ns, no significance.

To explore the impact of DAGs expression on BC, we performed clustering on 874 samples from the TCGA cohort based on these 24 DAGs using the ConsensusClusterPlus package. The Delta area curve and the cumulative distribution function (CDF) curve indicated that two clusters related to disulfidptosis were the most suitable (**Figure 3A**). The heatmap from clustering displayed that Cluster 1 had a larger proportion of DAGs, and Cluster 1 corresponded to a poorer prognosis (**Figures 3B, C**). The Sankey plot illustrated the overall distribution of two clusters in immune cell subtypes (**Figure 3D**). The box plot revealed that Macrophages M2, T cells CD4 memory resting, and Mast cells resting were more abundant on cluster 1, whereas T cells regulatory (Tregs), T cells CD8, Plasma cells, T cells follicular helper, and B cells naive were more abundant on cluster 2 (**Figure 3E**).

### 4.4 Predictive model building and validation

**Figure 4:**
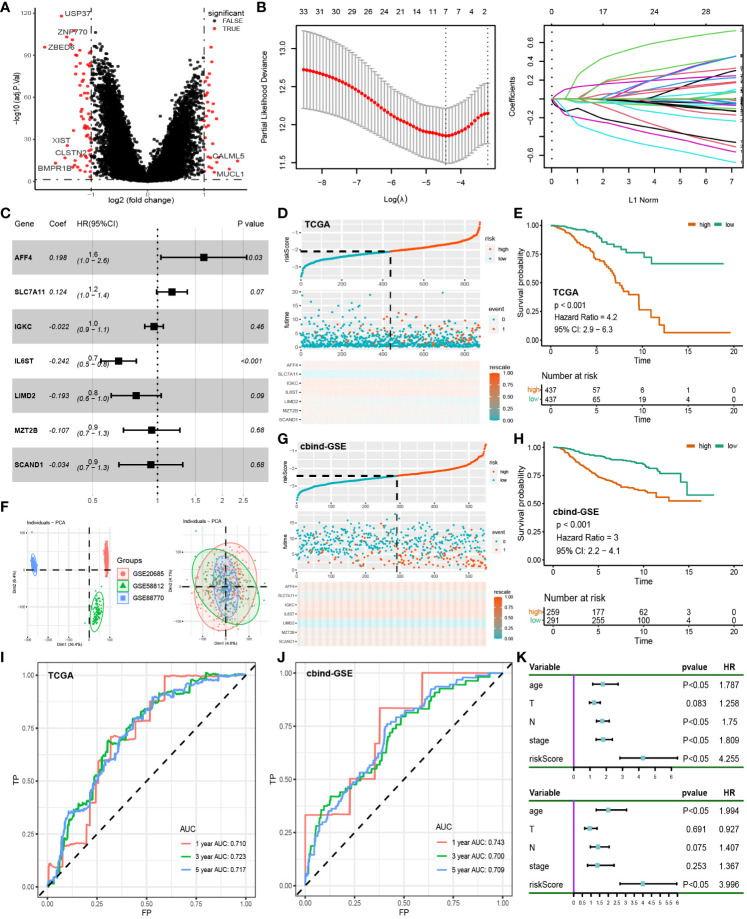
Construction of prognostic models: **(A)** Differential analysis of disulfidptosis-related clusters in the TCGA cohort. **(B)** Lasso regression was used to reduce the dimensionality of differential genes. **(C)** Multifactorial Cox regression of prognosis-related genes. **(D)** Risk plot of the training cohort. **(E)** Survival analysis of the training cohort. **(F)** Removal of batch effect in GSE cohorts, from Dim1 (36.4%) to Dim1 (4.6%). **(G)** Risk plot of the test cohort. **(H)** Survival analysis of the test cohort. **(I)** ROC curve of the training cohort. **(J)** ROC curve of the test cohort. **(K)** Univariate and multivariate Cox regression of clinical characteristics and riskscore.

Conducting differential analysis between two clusters, under the criteria of p-value < 0.05 and |logFC| > 1, we identified 93 differentially expressed genes associated with disulfidptosis (**Figure 4A**). First, univariate Cox regression identified 33 genes (**Supplementary Table S5**), and after dimension reduction using Lasso regression, 7 genes were obtained (**Figure 4B**). Then, a prognostic model was built using these genes (**Figure 4C**), and the riskscore was estimated as follows: AFF4 * 0.198 + SLC7A11 * 0.124 + IGKC * -0.022 + IL6ST * -0.242 + LIMD2 * -0.193 + MAT2B * -0.107 + SCAND1 * -0.034. According to the average riskscore, patients were divided into high-risk group and low-risk group. The risk plot and survival analysis demonstrated that the high-risk group had more death cases and corresponded to a worse prognosis (**Figures 4D, E**). The AUC value of the ROC curve showed a good prediction of survival (1, 3, 5-year: 0.710, 0.723, 0.717) (**Figure 4I**). The sva package was utilized to merge the GSE20685, GSE58812, and GSE88770 datasets into a cbind-GSE for external validation (**Figure 4F**). The distribution of deceased cases and survival analysis results from cbind-GSE analysis were consistent with the observations in the training cohort (**Figures 4G, H**), demonstrating good AUC values (1, 3, 5 years: 0.743, 0.700, 0.709) (**Figure 4J**). This indicates that our model has good accuracy and reliability in predicting prognosis. Finally, through univariate and multivariate Cox analysis combining clinical features and riskscore, the riskscore was identified as an independent prognostic factor for BC patients (**Figure 4K**).

### 4.5 Nomogram creation, clinical characteristics in relation to riskscore

**Figure 5:**
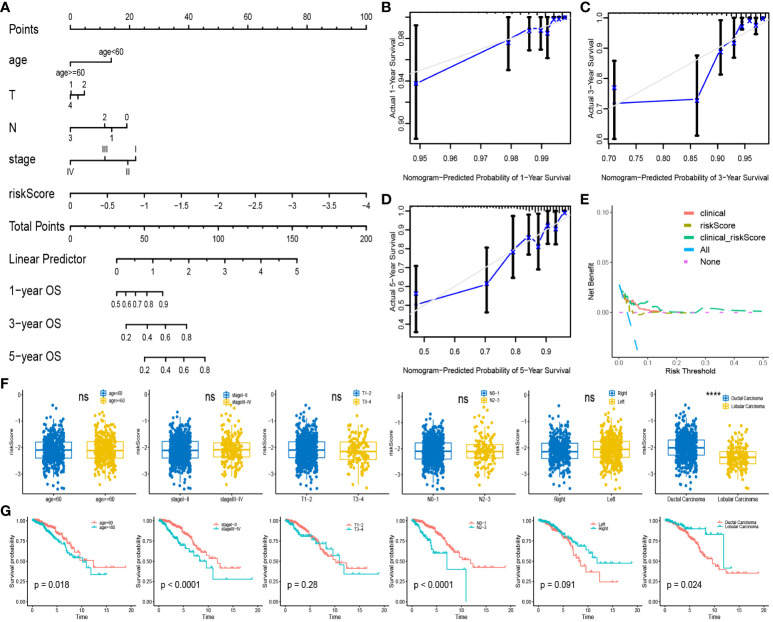
Nomogram and clinical Correlation Analysis: **(A)** Combine clinical features and riskscore to build nomogram. **(B–D)** Calibration curves for 1,3,5-year survival rates. **(E)** Clinical decision curve. **(F)** Expression of riskscore between different subgroups of clinical characteristics. **(G)** Survival analysis curves between subgroups with different clinical characteristics. ****P < 0.0001; ns, no significance.

We incorporated clinical characteristics to produce a nomogram, thus enhancing the clinical applicability of our model (**Figure 5A**). The high accuracy of survival prediction was revealed by calibration curves and DCA, indicating that the model has valid clinical decision-making capabilities (**Figures 5B–E**). Correlation analysis was performed among age, stage, T level, N level, subdivision, and tumor type with riskscore. The results revealed a significant difference only in the riskscore associated with tumor types, with ductal carcinoma having a higher riskscore than lobular carcinoma (**Figure 5F**). Furthermore, in survival analysis, older age, advanced stage, higher N level, and ductal carcinoma were all associated with poorer prognosis (**Figure 5G**).

### 4.6 Pathway enrichment analysis and somatic mutations

**Figure 6:**
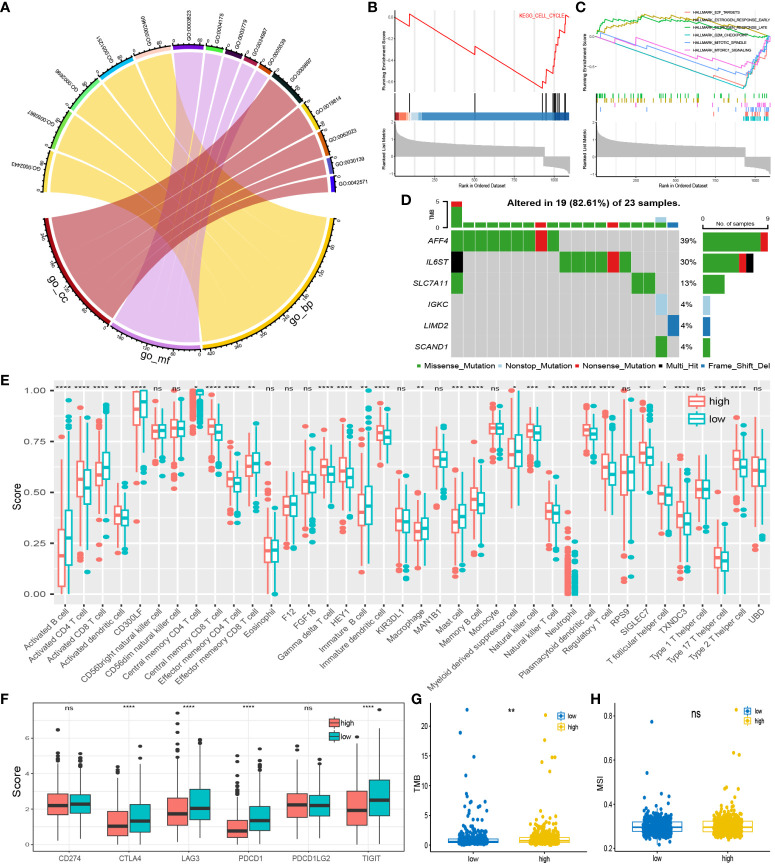
Pathway enrichment and immuno-infiltration analysis: **(A)** Enrichment pathways for the top five GO analyses. **(B)** Pathways in KEGG that are inversely associated with high-risk group. **(C)** Up and down regulated pathways in GSEA analysis. **(D)** Somatic mutation distribution of prognosis-related genes. **(E)** infiltration in 28 immune cells in both risk groups. **(F)** Expression of high and low-risk group at immune checkpoints. **(G, H)** TMB and MSI scores between the two risk groups. *P < 0.05; **P < 0.01; ***P < 0.001; ****P < 0.0001; ns, no significance.

To further elucidate the biological mechanisms, we conducted a pathway enrichment analysis. GSEA analysis revealed upregulation of the early estrogen response and late estrogen response pathways in the high-risk group, while the E2F targets, G2M checkpoint, mTORC1 signaling, and mitotic spindle pathways were downregulated in the high-risk group (**Figure 6C**). The cell cycle pathway and riskscore were inversely correlated in KEGG analysis (**Figure 6B**). As for GO analysis, the distribution of the top 5 pathways in biological process, molecular function, and cellular component is shown in the chord diagram (**Figure 6A**). Among them, the high-risk group exhibited the highest upregulation in leukocyte mediated immunity (GO:0002443), positive regulation of cell activation (GO:0050867), external side of the plasma membrane (GO:0009897), positive regulation of leukocyte activation (GO:0002696), and positive regulation of lymphocyte activation (GO:0051251). Furthermore, the waterfall plot of prognostic-related genes’ somatic mutations showed a relatively high mutation rate (82.61%) in the dataset of BC patients (**Figure 6D**). The genes with the most mutations were AFF4 and IL6ST, and the most frequent mutation type was Missense Mutation. It was worth noting that the high-risk group corresponds to a higher tumor mutation burden (TMB) (**Figure 6G**), while Microsatellite instability (MSI) scores did not differ significantly between these two groups (**Figure 6H**). This seems to suggest that BC patients in the high-risk group may exhibit a certain sensitivity to immunotherapy.

### 4.7 Immunity and drug sensitivity

**Figure 7:**
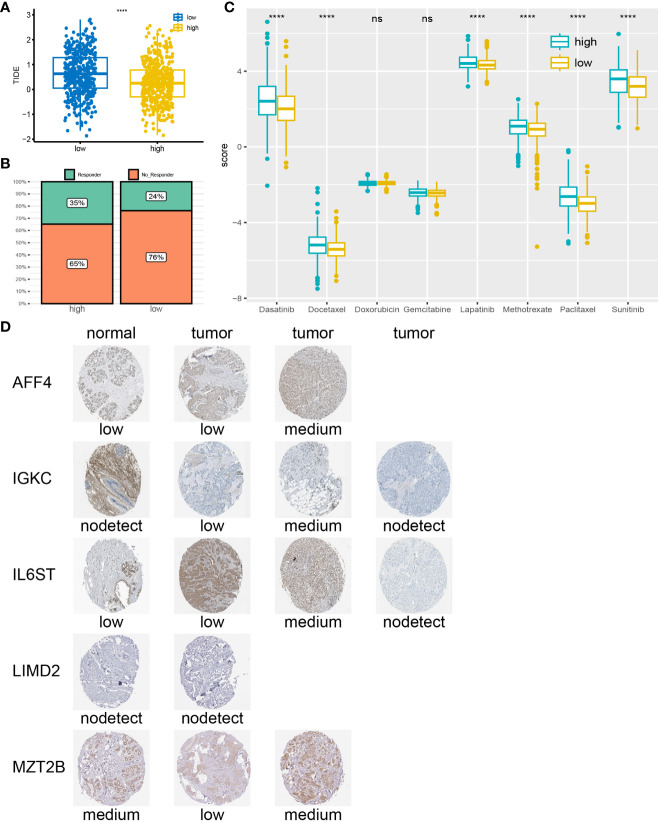
Drug sensitivity analysis and IHC: **(A)** High-risk group correspond to lower TIDE scores. **(B)** Higher percentage of immunotherapy response in high-risk group. **(C)** low-risk group may be more sensitive to Dasatinib, Docetaxel, Lapatinib, Methotrexate, Paclitaxel, and Sunitinib. **(D)** According to the IHC results obtained from the HPA website, the genes expression in tumor tissues is higher compared to normal tissues. ****P < 0.0001; ns, no significance.

Using the ssGSEA algorithm, we quantified 28 immune cell checkpoint markers in the dataset, revealing significant differences in multiple immune cells between these two groups (**Figure 6E**). The expression levels of CTLA4, LAG3, PDCD1 (PD-1), and TIGIT were higher in the low-risk group, indicating sensitivity to immunotherapy targeting these immune checkpoints (**Figure 6F**). Surprisingly, TIDE analysis showed lower TIDE values in the high-risk group, with a higher proportion of immunotherapy responders (**Figures 7A, B**). This suggests that, for some unknown reasons, immunotherapy is effective in the high-risk group as well. Additionally, drug sensitivity analysis revealed lower scores for dasatinib, docetaxel, lapatinib, methotrexate, paclitaxel, and sunitinib in the low-risk group, suggesting greater sensitivity of these drugs in patients from the low-risk group (**Figure 7C**).

### 4.8 Immunohistochemical validation of prognostic genes

We searched for 7 prognosis-related genes on the HPA website and ultimately retrieved the immunohistochemistry results for AFF4, IGKC, IL6ST, LIMD2, and MZT2B (**Figure 7D**). These genes exhibited different expression patterns between tumor tissues and normal tissues, categorized as “low,” “medium,” or “not detected,” indicating variations in gene expression among different individuals. However, it is noticeable that the expression levels of prognosis-related genes in tumor tissues are often higher than those in normal tissues.

## 5 Discussion

BC is a common disease worldwide and exhibits varying levels of prevalence in different regions (3). For instance, BC is the leading cancer among Chinese women, accounting for 9.6% of global mortality cases (25). In 2019, approximately 40,000 individuals in the United States lost their lives to BC (1). It’s not only women who are affected; male BC patients also face poor prognoses (26). The threat of BC to human health is undeniable. Despite multiple treatments, the survival rate of BC patients remains unsatisfactory, particularly for women with recurrent or advanced-stage metastatic breast cancer (27). In recent years, there has been an increasing awareness of the significant relevance of tumor-immune cell interactions in BC treatment, and a growing body of research has been dedicated to exploring this aspect (28, 29).

PCD is an essential process for normal cell turnover and maintaining homeostasis, and its regulation holds significant potential in cancer therapy (30, 31). Liu et al. proposed that tumor cells with high expression of SLC7A11, under glucose deprivation conditions, may undergo cell death (disulfidptosis) due to the accumulation of disulfides, providing a hopeful breakthrough for cancer treatment (9). However, several studies have suggested that the overexpression of SLC7A11 can promote tumor progression by inhibiting ferroptosis, contrary to Liu’s hypothesis (32, 33). Our analysis of single-cell data for BC revealed that ACTB and MYL6 had the highest expression levels in eight BC cell types, while SLC7A11 exhibited specific expression in MSCs. Aberrant expression of ACTB and MYL6 is associated with invasion and metastasis in many cancers (34, 35). Interestingly, Lin et al. found that MSCs and MSC-derived exosomes (MSC-Exo) could inhibit ferroptosis by maintaining the function of SLC7A11 (36). Hong et al. discovered that exosomes derived from umbilical cord mesenchymal stem cells (UC-MSC) could enhance SLC7A11 expression through sponge-like miR-494, thereby inhibiting ferroptosis (37). Our analysis indicates specific expression of SLC7A11 in MSCs, suggesting that MSCs or MSC-Exo may promote tumor cell disulfidptosis by enhancing SLC7A11 expression or function, potentially offering a novel direction for effective BC treatment. Further research is needed to explore the underlying mechanisms in this potential avenue of BC therapy.

Based on the DAGs expression profiles in the BC cohort, we employed an unsupervised clustering method to classify samples into two clusters. Cluster 1 exhibited higher DAGs abundance and corresponded to a poorer prognosis. Additionally, immune cell infiltration analysis revealed a higher abundance of mast cells and macrophages in Cluster 1, while Cluster 2 showed higher levels of CD8 T cells. These findings align with results observed in other cancer studies. Macrophages can inhibit T cell recruitment and function, promoting tumor initiation and malignant progression (38, 39). Mast cells are associated with tumor invasion, while elevated levels of CD8 T cells correspond to better prognosis in melanoma patients (40, 41). This suggests that the expression of DAGs may be correlated with the progression and lower survival rates of BC.

To further investigate the association between disulfidptosis and BC, we conducted a differential analysis between Cluster 1 and Cluster 2. Significant differentially expressed genes were filtered through Cox and Lasso regression, resulting in the identification of seven genes: AFF4, SLC7A11, IGKC, IL6ST, LIMD2, MAT2B, and SCAND1. Based on these genes, we constructed a prognosis model associated with disulfidptosis. Survival analysis revealed a shorter OS in the high-risk group. ROC curves demonstrated the reliability of our model, and it has passed external data validation. Furthermore, we developed a nomogram to enhance the clinical applicability of the model. Calibration curves and DCA indicated strong predictive performance and effective clinical utility of the model. It is noteworthy that there is a significant difference in the riskscore between ductal carcinoma and lobular carcinoma, with ductal carcinoma being associated with a shorter OS. Some researchers have proposed that invasive lobular carcinoma (ILC) has a favorable biological phenotype and better prognosis compared to invasive ductal carcinoma (IDC) (42, 43). However, studies have revealed that both the two correspond to almost the same prognosis (42). The clinical outcomes of IDC and ILC are influenced by histological subtypes, molecular subtypes, and Oestrogen receptors (43, 44). This suggests that there may be a correlation between disulfidaptosis and BC subtypes, and exploring the biological mechanisms behind it can provide guidance for the treatment of different subtypes of BC.

In recent decades, there have been continuous advancements in immunotherapy, which have facilitated in-depth research on the immune microenvironment of BC (45). Currently, more than 200 clinical trials of immunotherapies such as immune checkpoint inhibitors (ICIs) are underway (46). CTLA4/PD-1 therapeutic antibodies have already been sanctioned for melanoma, non-small cell lung cancer, and kidney cancer (47). The combined inhibition of CTLA4/PD-1 has proven effective in many diseases (48). Meanwhile, novel immune inhibitors such as LAG3/TIGIT are being extensively studied (49). When we applied immune cell infiltration to our model, the results showed higher expression of CTLA4, LAG3, PDCD1 (PD-1), and TIGIT in the low-risk group. CTLA4/PD-1 and LAG3/TIGIT immune inhibitors may be more effective in treating BC patients in the low-risk group. Surprisingly, the high-risk group corresponds to a higher TMB score and a lower TIDE score, with a higher response rate to immunotherapy. TMB has been demonstrated as a biological marker for the effectiveness of immunotherapy in many cancers, while a high TIDE score represents a low response to immunotherapy (50, 51). This suggests that immunotherapy is effective in the high-risk group as well. The impact of genes on a tumor’s response to immunotherapy remains a vague question (52). Despite the success of immunotherapies like CTLA4/PD-1, there are still many unknown immune checkpoints to be discovered. New candidate ICIs such as Serpinb 9 and Adam 2 are currently being explored by researchers (52–54). We speculate that, in addition to known immune checkpoints like CTLA4/PD-1 and LAG3/TIGIT, the high expression of unknown novel ICIs in the high-risk group might contribute to this phenomenon. Elucidating the potential biological mechanisms underlying this could significantly aid in the selection of immunotherapy for BC patients. Additionally, drug sensitivity analysis indicated that Dasatinib, Docetaxel, Lapatinib, Methotrexate, Paclitaxel, and Sunitinib may be more effective in low-risk group patients.

Lastly, a search was conducted on the HPA website for IHC results of prognostic-related genes. It was found that the expression levels in normal tissues and tumor tissues differed, possibly due to individual variations. Overall, the abundance of prognosis-related genes in tumor tissues is higher than in normal tissues, further validating the reliability of our model.

In this study, we compared the abundance of DAGs in different cells of BC and identified the specific expression of SLC7A11 in MSCs. Based on the expression profiles of DAGs, we classified BC patients into two clusters and performed differential analysis between the clusters. Genes showing significant differences were further selected through Cox and Lasso regression, resulting in seven genes used to construct a prognosis model related to disulfidptosis. This model accurately predicts the OS of BC patients and has been validated externally. Subsequently, we explored pathway enrichment, immune therapy efficacy, and chemotherapy drug sensitivity. The results indicated higher sensitivity to chemotherapy drugs in the low-risk group, while both groups showed potential applicability for immune therapy, requiring further mechanistic research for deeper exploration. However, our study has several limitations. Firstly, the proposition that MSCs may serve as a window for promoting cancer treatment through disulfidptosis is based solely on sequencing data analysis, necessitating extensive mechanistic studies to validate this result. Secondly, we only validated at the gene expression level through IHC, and the protein-level validation remains unknown. Lastly, the majority of study populations consist of Western or Caucasian individuals, and further research including more diverse ethnic groups is needed to validate our findings.

In summary, this study conducted a comprehensive exploration of the relationship between disulfidptosis and BC, and established an effective prognostic prediction model. We found that patients in both high-risk and low-risk groups may respond to immunotherapy, requiring further mechanistic research for clarification. Additionally, we revealed the significant potential of MSCs in promoting disulfidptosis, providing inspiration for subsequent studies. Overall, our research could offer more personalized guidance for the prognosis prediction and treatment selection of BC patients.

## Data availability statement

Publicly available datasets were analyzed in this study. This data can be found here: TCGA-BRCA from https://portal.gdc.cancer.gov/; GSE20685, GSE58812, GSE88770, and GSE176078 from https://www.ncbi.nlm.nih.gov/gds/.

## Author contributions

XC: Data curation, Project administration, Resources, Visualization, Writing – original draft. GH: Funding acquisition, Supervision, Writing – review & editing. QY: Writing – review & editing.

## Supplementary material

Supplementary Table 1 The detail of all cohorts in our study.

Supplementary Table 2 The detail of all cohorts in our study.

Supplementary Table 3 The detail of all cohorts in our study.

Supplementary Table 4 The detail of all cohorts in our study.

Supplementary Table 5 List of genes screened by univariate Cox regression.

## References

[1] Siegel RL Miller KD Jemal A. Cancer statistics, 2020. CA Cancer J Clin (2020) 70:7–30. doi: 10.3322/caac.21590 31912902

[2] Veronesi U Boyle P Goldhirsch A Orecchia R Viale G. Breast cancer. Lancet (2005) 365:1727–41. doi: 10.1016/S0140-6736(05)66546-4 15894099

[3] Harbeck N Gnant M. Breast cancer. Lancet (2017) 389:1134–50. doi: 10.1016/S0140-6736(16)31891-8 27865536

[4] Dent R Trudeau M Pritchard KI Hanna WM Kahn HK Sawka CA. Triple-negative breast cancer: clinical features and patterns of recurrence. Clin Cancer Res (2007) 13:4429–34. doi: 10.1158/1078-0432.CCR-06-3045 17671126

[5] Liang Y Zhang H Song X Yang Q. Metastatic heterogeneity of breast cancer: Molecular mechanism and potential therapeutic targets. Semin Cancer Biol (2020) 60:14–27. doi: 10.1016/j.semcancer.2019.08.012 31421262

[6] Tong X Tang R Xiao M Xu J Wang W Zhang B. Targeting cell death pathways for cancer therapy: recent developments in necroptosis, pyroptosis, ferroptosis, and cuproptosis research. J Hematol Oncol (2022) 15:174. doi: 10.1186/s13045-022-01392-3 36482419 PMC9733270

[7] Peng F Liao M Qin R Zhu S Peng C Fu L. Regulated cell death (RCD) in cancer: key pathways and targeted therapies. Signal Transduct Target Ther (2022) 7:286. doi: 10.1038/s41392-022-01110-y 35963853 PMC9376115

[8] Ketelut-Carneiro N Fitzgerald KA. Apoptosis, pyroptosis, and necroptosis-oh my! The many ways a cell can die. J Mol Biol (2022) 434:167378. doi: 10.1016/j.jmb.2021.167378 34838807

[9] Liu X Nie L Zhang Y Yan Y Wang C Colic M. Actin cytoskeleton vulnerability to disulfide stress mediates disulfidptosis. Nat Cell Biol (2023) 25:404–14. doi: 10.1038/s41556-023-01091-2 PMC10027392 36747082

[10] Liu X Olszewski K Zhang Y Lim EW Shi J Zhang X. Cystine transporter regulation of pentose phosphate pathway dependency and disulfide stress exposes a targetable metabolic vulnerability in cancer. Nat Cell Biol (2020) 22:476–86. doi: 10.1038/s41556-020-0496-x PMC7194135 32231310

[11] Joly JH Delfarah A Phung PS Parrish S Graham NA. A synthetic lethal drug combination mimics glucose deprivation-induced cancer cell death in the presence of glucose. J Biol Chem (2020) 295:1350–65. doi: 10.1074/jbc.RA119.011471 PMC6996897 31914417

[12] Carlisle AE Lee N Matthew-Onabanjo AN Spears ME Park SJ Youkana D. Selenium detoxification is required for cancer-cell survival. Nat Metab (2020) 2:603–11. doi: 10.1038/s42255-020-0224-7 PMC7455022 32694795

[13] Xu L Wang S Zhang D Wu Y Shan J Zhu H. Machine learning- and WGCNA-mediated double analysis based on genes associated with disulfidptosis, cuproptosis and ferroptosis for the construction and validation of the prognostic model for breast cancer. J Cancer Res Clin Oncol (2023) 149:16511–23. doi: 10.1007/s00432-023-05378-7 37712959 PMC11798189

[14] Wu SZ Al-Eryani G Roden DL Junankar S Harvey K Andersson A. A single-cell and spatially resolved atlas of human breast cancers. Nat Genet (2021) 53:1334–47. doi: 10.1038/s41588-021-00911-1 PMC9044823 34493872

[15] Li T Chen Z Wang Z Lu J Chen D. Combined signature of N7-methylguanosine regulators with their related genes and the tumor microenvironment: a prognostic and therapeutic biomarker for breast cancer. Front Immunol (2023) 14:1260195. doi: 10.3389/fimmu.2023.1260195 37868988 PMC10585266

[16] Zou Y Xie J Zheng S Liu W Tang Y Tian W. Leveraging diverse cell-death patterns to predict the prognosis and drug sensitivity of triple-negative breast cancer patients after surgery. Int J Surg (2022) 107:106936. doi: 10.1016/j.ijsu.2022.106936 36341760

[17] Butler A Hoffman P Smibert P Papalexi E Satija R. Integrating single-cell transcriptomic data across different conditions, technologies, and species. Nat Biotechnol (2018) 36:411–20. doi: 10.1038/nbt.4096 PMC6700744 29608179

[18] Becht E McInnes L Healy J Dutertre C-A Kwok IWH Ng LG. Dimensionality reduction for visualizing single-cell data using UMAP. Nat Biotechnol (2018). doi: 10.1038/nbt.4314 30531897

[19] Grün D van Oudenaarden A. Design and analysis of single-cell sequencing experiments. Cell (2015) 163:799–810. doi: 10.1016/j.cell.2015.10.039 26544934

[20] Wilkerson MD Hayes DN. ConsensusClusterPlus: a class discovery tool with confidence assessments and item tracking. Bioinformatics (2010) 26:1572–3. doi: 10.1093/bioinformatics/btq170 PMC2881355 20427518

[21] Le T Aronow RA Kirshtein A Shahriyari L. A review of digital cytometry methods: estimating the relative abundance of cell types in a bulk of cells. Brief Bioinform (2021) 22:bbaa219. doi: 10.1093/bib/bbaa219 33003193 PMC8293826

[22] Ritchie ME Phipson B Wu D Hu Y Law CW Shi W. limma powers differential expression analyses for RNA-sequencing and microarray studies. Nucleic Acids Res (2015) 43:e47. doi: 10.1093/nar/gkv007 25605792 PMC4402510

[23] Yu G Wang L-G Han Y He Q-Y. clusterProfiler: an R package for comparing biological themes among gene clusters. OMICS: A J Integr Biol (2012) 16:284–7. doi: 10.1089/omi.2011.0118 PMC3339379 22455463

[24] Yang W Soares J Greninger P Edelman EJ Lightfoot H Forbes S. Genomics of Drug Sensitivity in Cancer (GDSC): a resource for therapeutic biomarker discovery in cancer cells. Nucleic Acids Res (2013) 41:D955–61. doi: 10.1093/nar/gks1111 PMC3531057 23180760

[25] Fan L Strasser-Weippl K Li J-J St Louis J Finkelstein DM Yu K-D. Breast cancer in China. Lancet Oncol (2014) 15:e279–289. doi: 10.1016/S1470-2045(13)70567-9 24872111

[26] Donegan WL. Cancer of the breast in men. CA Cancer J Clin (1991) 41:339–54. doi: 10.3322/canjclin.41.6.339 1933534

[27] Maughan KL Lutterbie MA Ham PS. Treatment of breast cancer. Am Fam Phys (2010) 81:1339–46. 20521754

[28] Hammerl D Smid M Timmermans AM Sleijfer S Martens JWM Debets R. Breast cancer genomics and immuno-oncological markers to guide immune therapies. Semin Cancer Biol (2018) 52:178–88. doi: 10.1016/j.semcancer.2017.11.003 29104025

[29] Emens LA. Breast cancer immunotherapy: facts and hopes. Clin Cancer Res (2018) 24:511–20. doi: 10.1158/1078-0432.CCR-16-3001 PMC5796849 28801472

[30] Tower J. Programmed cell death in aging. Ageing Res Rev (2015) 23:90–100. doi: 10.1016/j.arr.2015.04.002 25862945 PMC4480161

[31] Christgen S Tweedell RE Kanneganti T-D. Programming inflammatory cell death for therapy. Pharmacol Ther (2022) 232:108010. doi: 10.1016/j.pharmthera.2021.108010 34619283 PMC8930427

[32] Koppula P Zhuang L Gan B. Cystine transporter SLC7A11/xCT in cancer: ferroptosis, nutrient dependency, and cancer therapy. Protein Cell (2021) 12:599–620. doi: 10.1007/s13238-020-00789-5 33000412 PMC8310547

[33] Lang X Green MD Wang W Yu J Choi JE Jiang L. Radiotherapy and immunotherapy promote tumoral lipid oxidation and ferroptosis via synergistic repression of SLC7A11. Cancer Discov (2019) 9:1673–85. doi: 10.1158/2159-8290.CD-19-0338 PMC6891128 31554642

[34] Gu Y Tang S Wang Z Cai L Lian H Shen Y. A pan-cancer analysis of the prognostic and immunological role of β-actin (ACTB) in human cancers. Bioengineered (2021) 12:6166–85. doi: 10.1080/21655979.2021.1973220 PMC8806805 34486492

[35] Vierthaler M Sun Q Wang Y Steinfass T Poelchen J Hielscher T. ADCK2 knockdown affects the migration of melanoma cells via MYL6. Cancers (Basel) (2022) 14:1071. doi: 10.3390/cancers14041071 35205819 PMC8869929

[36] Lin F Chen W Zhou J Zhu J Yao Q Feng B. Mesenchymal stem cells protect against ferroptosis via exosome-mediated stabilization of SLC7A11 in acute liver injury. Cell Death Dis (2022) 13:271. doi: 10.1038/s41419-022-04708-w 35347117 PMC8960810

[37] Hong T Zhao T He W Xia J Huang Q Yang J. Exosomal circBBS2 inhibits ferroptosis by targeting miR-494 to activate SLC7A11 signaling in ischemic stroke. FASEB J (2023) 37:e23152. doi: 10.1096/fj.202300317RRR 37603538

[38] DeNardo DG Ruffell B. Macrophages as regulators of tumour immunity and immunotherapy. Nat Rev Immunol (2019) 19:369–82. doi: 10.1038/s41577-019-0127-6 PMC7339861 30718830

[39] Cassetta L Pollard JW. Targeting macrophages: therapeutic approaches in cancer. Nat Rev Drug Discov (2018) 17:887–904. doi: 10.1038/nrd.2018.169 30361552

[40] Majorini MT Colombo MP Lecis D. Few, but efficient: the role of mast cells in breast cancer and other solid tumors. Cancer Res (2022) 82:1439–47. doi: 10.1158/0008-5472.CAN-21-3424 PMC9306341 35045983

[41] Zhu G Su H Johnson CH Khan SA Kluger H Lu L. Intratumour microbiome associated with the infiltration of cytotoxic CD8+ T cells and patient survival in cutaneous melanoma. Eur J Cancer (2021) 151:25–34. doi: 10.1016/j.ejca.2021.03.053 33962358 PMC8184628

[42] Arpino G Bardou VJ Clark GM Elledge RM. Infiltrating lobular carcinoma of the breast: tumor characteristics and clinical outcome. Breast Cancer Res (2004) 6:R149–156. doi: 10.1186/bcr767 PMC400666 15084238

[43] Adachi Y Ishiguro J Kotani H Hisada T Ichikawa M Gondo N. Comparison of clinical outcomes between luminal invasive ductal carcinoma and luminal invasive lobular carcinoma. BMC Cancer (2016) 16:248. doi: 10.1186/s12885-016-2275-4 27015895 PMC4807554

[44] Mhuircheartaigh JN Curran C Hennessy E Kerin MJ. Prospective matched-pair comparison of outcome after treatment for lobular and ductal breast carcinoma. Br J Surg (2008) 95:827–33. doi: 10.1002/bjs.6042 18498127

[45] Dieci MV Miglietta F Guarneri V. Immune infiltrates in breast cancer: recent updates and clinical implications. Cells (2021) 10:223. doi: 10.3390/cells10020223 33498711 PMC7911608

[46] Onkar SS Carleton NM Lucas PC Bruno TC Lee AV Vignali DAA. The great immune escape: understanding the divergent immune response in breast cancer subtypes. Cancer Discov (2023) 13:23–40. doi: 10.1158/2159-8290.CD-22-0475 36620880 PMC9833841

[47] Topalian SL Taube JM Anders RA Pardoll DM. Mechanism-driven biomarkers to guide immune checkpoint blockade in cancer therapy. Nat Rev Cancer (2016) 16:275–87. doi: 10.1038/nrc.2016.36 PMC5381938 27079802

[48] Anderson AC Joller N Kuchroo VK. Lag-3, tim-3, and TIGIT: co-inhibitory receptors with specialized functions in immune regulation. Immunity (2016) 44:989–1004. doi: 10.1016/j.immuni.2016.05.001 27192565 PMC4942846

[49] Kraehenbuehl L Weng C-H Eghbali S Wolchok JD Merghoub T. Enhancing immunotherapy in cancer by targeting emerging immunomodulatory pathways. Nat Rev Clin Oncol (2022) 19:37–50. doi: 10.1038/s41571-021-00552-7 34580473

[50] Chan TA Yarchoan M Jaffee E Swanton C Quezada SA Stenzinger A. Development of tumor mutation burden as an immunotherapy biomarker: utility for the oncology clinic. Ann Oncol (2019) 30:44–56. doi: 10.1093/annonc/mdy495 30395155 PMC6336005

[51] Jiang P Gu S Pan D Fu J Sahu A Hu X. Signatures of T cell dysfunction and exclusion predict cancer immunotherapy response. Nat Med (2018) 24:1550–8. doi: 10.1038/s41591-018-0136-1 PMC6487502 30127393

[52] Dervovic D Malik AA Chen ELY Narimatsu M Adler N Afiuni-Zadeh S. *In vivo* CRISPR screens reveal Serpinb9 and Adam2 as regulators of immune therapy response in lung cancer. Nat Commun (2023) 14:3150. doi: 10.1038/s41467-023-38841-7 37258521 PMC10232477

[53] Jiang L Wang Y-J Zhao J Uehara M Hou Q Kasinath V. Direct tumor killing and immunotherapy through anti-serpinB9 therapy. Cell (2020) 183:1219–1233.e18. doi: 10.1016/j.cell.2020.10.045 33242418 PMC7927154

[54] Han R Yu L Zhao C Li Y Ma Y Zhai Y. Inhibition of SerpinB9 to enhance granzyme B-based tumor therapy by using a modified biomimetic nanoplatform with a cascade strategy. Biomaterials (2022) 288:121723. doi: 10.1016/j.biomaterials.2022.121723 35963816

